# Quantitative Assessment of Liver Fibrosis: B1 Inhomogeneity-Corrected VFA T1 Mapping on Gadobenate Dimeglumine-Enhanced MRI

**DOI:** 10.2174/0115734056393310250929071043

**Published:** 2025-10-20

**Authors:** Lianbang Wang, Hui Ma, Xiao Feng, Zijian Shen, Jin Cui, Ximing Wang, Gongzheng Wang, Xinya Zhao

**Affiliations:** 1 Department of Radiology, Shandong Provincial Hospital Affiliated to Shandong First Medical University, No. 324, Jingwu Road, Jinan 250021, Shandong Province, China; 2 Department of Radiology, The First Affiliated Hospital of Fujian Medical University, Fuzhou, Fujian Province, China; 3 Department of Gastroenterology, Shandong Provincial Hospital Affiliated to Shandong First Medical University, No. 324, Jingwu Road, Jinan 250021, Shandong Province, China; 4 Department of Radiology, Liaocheng People's Hospital, Liaocheng, 252000, Shandong Province, China; 5 Key Laboratory of Endocrine Glucose & Lipids Metabolism and Brain Aging, Shandong First Medical University, Ministry of Education, Jinan, China

**Keywords:** Gadobenic acid, Magnetic resonance imaging, Liver fibrosis, T1 mapping, Organic anion transporters, Variable Flip Angle

## Abstract

**Introduction:**

Accurate early diagnosis and assessment of liver fibrosis are important for patient treatment and prognosis. This study explored the value of Gd-BOPTA-enhanced T1 mapping *via* the B1 inhomogeneity-corrected Variable Flip Angle (VFA) method for staging liver fibrosis in rats.

**Methods:**

Sprague‒Dawley rats were divided into one control group (n = 6) and four carbon tetrachloride-induced liver fibrosis groups (n = 6 each group). T1 mapping *via* B1 inhomogeneity-corrected VFA was performed before and 90 minutes after Gd-BOPTA administration. Precontrast T1 values (T1_pre_), postcontrast T1 values (T1_post_), and the reduction rate of T1 values (ΔT1%) were quantified on T1 mapping images. The diagnostic performance was evaluated by the Area Under the Receiver Operating Characteristic Curve (AUC). The correlations between T1_pre_, T1_post_, ΔT1% values, and the expression levels of hepatocyte transporters (Oatp1a1 and Mrp2) were evaluated.

**Results:**

T1_post_ and ΔT1% were significantly correlated with liver fibrosis stage (r = 0.832, *p* < 0.001; r = −0.798, *p* < 0.001, respectively), whereas T1_pre_ was not significantly correlated with fibrosis stage (r = 0.357, *p* = 0.062). The AUCs of T1_post_ and ΔT1% were greater than those of postcontrast signal intensity for diagnosing stages F2–F4 (0.936, 0.941 *vs.* 0.791; *p* = 0.043, 0.038, respectively), F3–F4 (0.928, 0.861 *vs.* 0.660; *p* = 0.003, 0.028, respectively) and F4 (0.965, 0.896 *vs.* 0.761; *p* = 0.021, 0.049, respectively). Oatp1a1 and Mrp2 expression levels correlated significantly with T1_post_ (r = −0.859, *p =* 0.001; r = −0.697, *p* = 0.017) and ΔT1% (r = 0.891, *p* < 0.001; r = 0.685, *p* = 0.020), respectively.

**Discussion:**

T1_post_ and ΔT1% were significantly correlated with liver fibrosis stages, and have good diagnostic performance for staging liver fibrosis. The protein expression levels of Oatp1a1 and Mrp2 correlated significantly with T1_post_ and ΔT1%.

**Conclusion:**

Gd-BOPTA-enhanced T1 mapping *via* the B1 inhomogeneity-corrected VFA shows promise as a potentially accurate and reliable tool for quantifying liver fibrosis stages.

## 1 INTRODUCTION

Liver fibrosis is characterized by the pathological deposition of extracellular matrix proteins caused by chronic liver damage from diverse etiologies, such as viral hepatitis, alcoholic liver disease, and metabolic dysfunction-associated steatotic liver disease [1, 2]. As fibrosis progresses, it can lead to liver cirrhosis, hepatic failure, and even hepatocellular carcinoma [3]. The precision of fibrotic staging during clinical evaluation fundamentally determines the efficacy of therapeutic interventions and long-term prognostic outcomes.

Liver biopsy is recognized as the reference standard for diagnosing and staging liver fibrosis. However, it is invasive and suffers from intraobserver variability and sampling errors [4]. In recent years, several diagnostic methods, such as biochemical blood parameters [5], ultrasound elastography [6, 7], and magnetic resonance elastography [8], have been validated for staging hepatic fibrosis. However, ultrasound elastography is less sensitive in obese patients [9]. In addition, magnetic resonance elastography sequences may fail in patients with hepatic iron deposition [6, 10] and may not be widely available in patients with hepatic fibrosis due to their high cost. Therefore, developing simple and noninvasive diagnostic techniques for accurate staging of hepatic fibrosis remains a critical clinical priority.

Gadobenate dimeglumine (Gd-BOPTA, Bracco Imaging, Italy) is a widely used hepatobiliary-specific agent for diagnosing liver diseases [11]. Gd-BOPTA enters hepatocytes through organic anion transporting polypeptides (Oatps) and is excreted into bile *via* Multidrug resistance-associated proteins (Mrps) 40–120 minutes after administration [12]. In clinical practice, this agent is routinely utilized in the United States, China, Italy, and other regions for assessing liver function and detecting focal lesions [13, 14]. As liver fibrosis progresses, it leads to changes in the T1 relaxation time of the fibrotic tissue [2, 15]. T1 mapping is a reliable and quantitative method for determining the T1 relaxation time, which is a form of organizational representation based on the parameter method [16]. T1 mapping can be acquired through different methods. Unlike the conventional modified look-locker inversion recovery sequence, the B1 inhomogeneity-corrected Variable Flip Angle (VFA) T1 mapping technique can achieve whole-liver coverage within a short time and avoid spatial variations in the measured T1 values and flip angles [17, 18]. The measurement of T1 relaxation time by B1 inhomogeneity-corrected VFA T1 mapping may be more accurate for assessing liver fibrosis.

Our study aimed to explore the value of Gd-BOPTA-enhanced T1 mapping *via* the B1 inhomogeneity-corrected VFA method for staging liver fibrosis in a rat model, and the relationships between T1 mapping parameters and hepatocyte transporter expression levels were also studied.

## 2 METHODS

### 2.1 Animal Model

This study is an experimental study. A total of 30 male 8-week-old Sprague‒Dawley rats, weighing 260 ± 30 grams, were purchased from Beijing Weitong Lihua Experimental Animal Technology Co., Ltd. To induce liver fibrosis with different stages, 24 rats were randomly divided into 4 groups (n = 6 each group) and were intraperitoneally injected with a mixture of carbon tetrachloride (CCl_4_) and olive oil (CCl_4_: olive oil = 2:3) at a dose of 1.0 ml/kg twice a week for 2, 4, 6, or 8 weeks, respectively [19]. The distribution of liver fibrosis reflected the natural progression of fibrosis in the model, consistent with clinical chronic liver disease. The control group included 6 rats that received only pure olive oil for 8 weeks. The rats underwent liver T1 mapping scans and subsequent liver harvesting 3 days after the last CCl_4_ insult. Animals were handled in accordance with the US National Research Council's Guide for the Care and Use of Laboratory Animals. The experimental protocols were approved by Shandong Provincial Hospital Animal Care & Welfare Committee (No. 2021-087), affiliated with Shandong Provincial Hospital, Affiliated to Shandong First Medical University, China.

### 2.2 MRI Protocol

MRI was performed on a 3T system (Magnetom Prisma, Siemens Healthineers, Erlangen, Germany), using an 8-channel phased-array animal coil with a 5 cm inner diameter (Chenguang Medical Technologies Co.). The rats were anesthetized with tribromoethanol (320 mg/kg body weight) by intraperitoneal injection and placed in a prone position to reduce respiratory motion. The B1 mapping using FLASH sequence was acquired for T1 map correction using the following parameters: repetition time (TR) = 3680 ms, echo time (TE) = 2.22 ms, flip angle = 8°, field of view (FOV) = 300 × 244 mm^2^, slice thickness = 3 mm, matrix = 52 × 64, voxel size = 4.7 × 4.7 × 3.0 mm^3^, acquisition time = 7 s. The T1 mapping was acquired using a 3D gradient echo sequence with the Volumetric Interpolated Breath-hold Examination (VIBE) sequence using the following parameters: TE/TR = 2.87/5.96 ms, slice thickness = 3.0 mm, FOV = 100 × 82.5 mm^2^, matrix = 99 × 160, flip angle = 3° and 15°, bandwidth = 490 Hz/pixel, number of slices = 20, acquisition time = 2 minutes 31 seconds. T1 mapping was performed before and 90 minutes after Gd-BOPTA injection, which was injected *via* the tail vein at 1 ml/s, followed by flushing with 0.5 ml of saline. Following B1 field correction, parametric T1 maps were automatically generated in-line after data acquisition using the MapIt processing tool (Siemens Healthcare, Erlangen, Germany).

### 2.3 Imaging Analysis

**Fig. (1):**
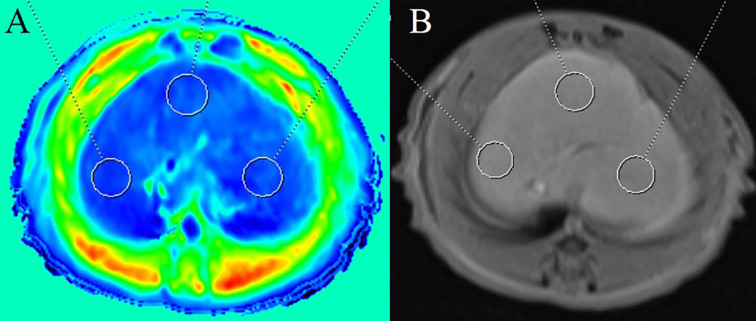
Examples of placing Regions of Interest (ROIs) in a rat model. Three ROIs are placed in the liver parenchyma on (**A**) Gd-BOPTA-enhanced T1 mapping images and (**B**) postcontrast gray images.

All image acquisition and analyses were performed in a double-blind manner by two experienced radiologists. The intraclass correlation coefficient was used to evaluate the repeatability. Image postprocessing was performed by Syngo *via* software (Siemens Healthineers). The T1 relaxation time on the T1 maps and the signal intensity on the postcontrast gray images were drawn freehand in the liver parenchyma using the Region of Interest (ROI). The ROI met the following criteria: (1) Avoidance of obvious focal lesions, main branch vessels, and abnormal perfusion; (2) the size and shape of the ROI should be the same or roughly the same; (3) three ROIs were placed in the right lobe, left lobe, and middle lobe of the liver, each with an area of approximately 15 mm^2^, and the average value from these three lobes was calculated (Fig. **1A**, **B**). The mean values of the ROIs of the two radiologists were considered representative of the T1 relaxation time. ΔT1% = (T1_pre_ − T1_post_)/T1_pre_ × 100%, where T1_pre_ is the precontrast T1 relaxation time, T1_post_ is the postcontrast T1 relaxation time, and ΔT1% is the reduction rate of the T1 values. SI_post_ represents the post-contrast signal intensity measured on T1-weighted images.

### 2.4 Histological Analysis

The rats in each group were humanely killed 8 hours after the completion of the MRI scan, and their livers were removed and subsequently fixed in 10% formalin buffered with phosphate. The samples of the rat livers were subjected to Masson’s trichrome staining. Two experienced pathologists, blinded to the MRI results, reviewed all the pathological specimens. In cases of disagreement, a final decision was reached through consensus. The remaining liver tissue was quickly cooled in liquid nitrogen and subsequently stored in a -80°C freezer for later protein extraction. On the basis of the METAVIR scoring system, liver fibrosis was staged as follows: F0, no fibrosis; F1, portal fibrosis without septa; F2, portal fibrosis with a few septa; F3, numerous septa without cirrhosis; F4, cirrhosis [20].

### 2.5 Western Blotting

The protein expression levels of Oatp1a1 and Mrp2 in the livers of the control group (stage F0) and cirrhosis group (stage F4) were determined *via* Western blot analysis. A monoclonal antibody against Oatp1 (Bioss, Beijing, China) and polyclonal antibodies against Mrp2 (Santa Cruz Biotechnology, Santa Cruz, CA, USA) were used. Densitometry was used to calculate the protein expression levels from the immunoreactive bands on the blot, which were reported as the ratio of the protein band intensity to that of the glyceraldehyde-3-phosphate dehydrogenase control.

### 2.6 Statistical Analysis

All the statistical analyses were performed with SPSS 25 (version 25.0) and MedCalc (version 20.0.1). The Shapiro‒Wilk test was used to analyze the normality of the data. One-way analysis of variance with a post hoc test was applied to compare the differences in T1_pre_, T1_post_, and ΔT1% among the different fibrosis stages. Pearson correlation analysis was applied to analyze the relationships between MRI quantitative parameters and liver fibrosis stages or the expression levels of hepatocyte transporters. The diagnostic performances of T1_pre_, T1_post_, ΔT1%, and SI_post_ for the liver fibrosis stages were compared *via* Areas Under the Receiver Operating Characteristic Curves (AUCs) and the DeLong test [16, 21]. Additionally, optimal cut-off values were determined *via* the Youden index, and the corresponding sensitivities and specificities were determined. All tests were two-sided, and *p* < 0.05 was regarded as statistically significant.

## 3 RESULTS

### 3.1 Pathological Staging of Liver Fibrosis in Rats

**Fig. (2):**
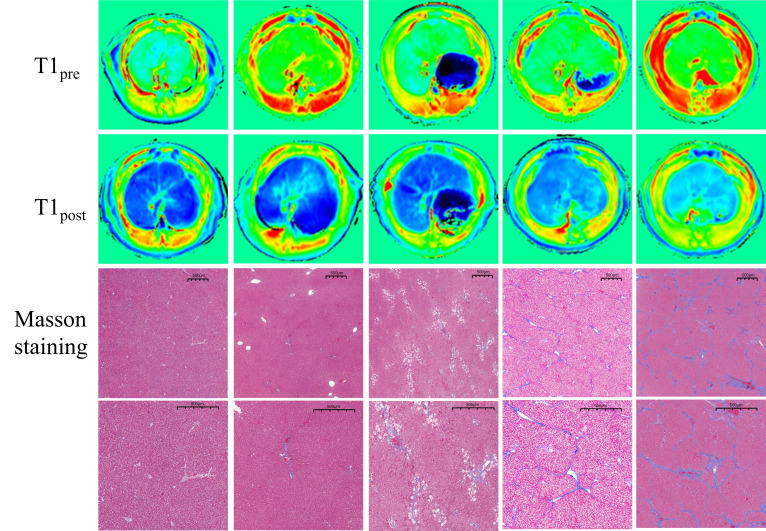
T1 maps of the rat liver and Masson’s trichrome staining of liver samples at stages F0–F4.

A total of 28 rats survived over the course of the MRI scan. Two rats died during the experiment and were not included in the final statistical analyses. The rats were killed 8 hours after completing the MRI scan. Pathologically, six rats were designated as having stage F0 fibrosis, five had stage F1, six had stage F2, six had stage F3, and five had stage F4 (Fig. **2**).

### 3.2 Differences in T1_pre_, T1_post_, and ΔT1% at Different Fibrosis Stages

**Fig. (3):**
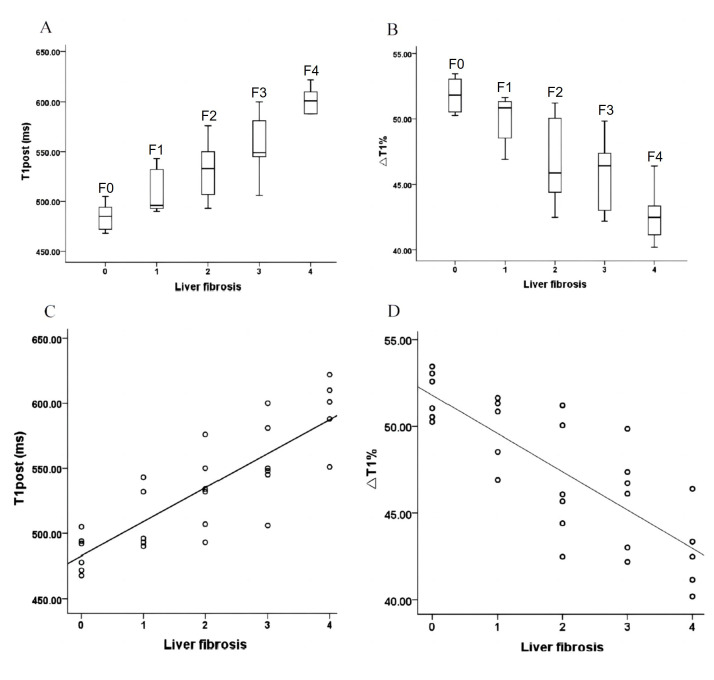
Boxplots of (**A**) T1_post_ and (**B**) ΔT1% in different fibrosis stages. Scatter plots showing the correlations between liver fibrosis stage and (**C**) T1_post_ (r = 0.832, *p* < 0.001) and (**D**) ΔT1% (r = −0.798, *p* < 0.001). T1_post_, postcontrast T1 relaxation time; ΔT1%, reduction rate of T1 relaxation time.

The intraclass correlation coefficient values of T1_pre_, T1_post_, ΔT1%, and SI_post_ were 0.901, 0.987, 0.931, and 0.943, respectively, indicating excellent agreement. The means and standard deviations (SDs) of T1_pre_, T1_post_, and ΔT1% are provided in Table **1**. There were significant differences in T1_post_ and ΔT1% among the different stages of liver fibrosis (all *p* < 0.05), and no significant differences in T1_pre_ were found among the different stages of liver fibrosis (*p* = 0.120). In the comparison of T1_post_ at different fibrosis stages, each pair of comparisons was significantly different (LSD post hoc tests, *p* < 0.05), except for stages F0 and F1 (484.83 ± 14.40 *vs.* 510.8 ± 24.77, *p* = 0.119) and stages F1 and F2 (510.8 ± 24.77 *vs.* 532 ± 29.70, *p* = 0.146) (Fig. **3A**). ΔT1% was not significantly different between stages F0 and F1 (51.82 ± 1.37 *vs.* 49.85 ± 2.05, *p* = 0.208) or stages F2 and F3 (46.65 ± 3.35 *vs.* 45.88 ± 2.85, *p* = 0.601), but significant differences were found in the other comparison groups (*p* < 0.05) (Fig. **3B**). T1_pre_ was significantly different between stages F2 and F4 (998.34 ± 43.09 *vs.* 1037.6 ± 14.43, *p* = 0.018) and was not significantly different in the other comparison groups (*p* > 0.05). T1_post_ and ΔT1% were significantly positively (r = 0.832, *p* < 0.001, Fig. **3C**) and negatively (r = −0.798, *p* < 0.001; Fig. **3D**), respectively, correlated with liver fibrosis stage, whereas T1_pre_ was not significantly correlated with the stage of liver fibrosis (r = 0.357, *p* = 0.062).

### 3.3 ROC Analysis

**Fig. (4):**
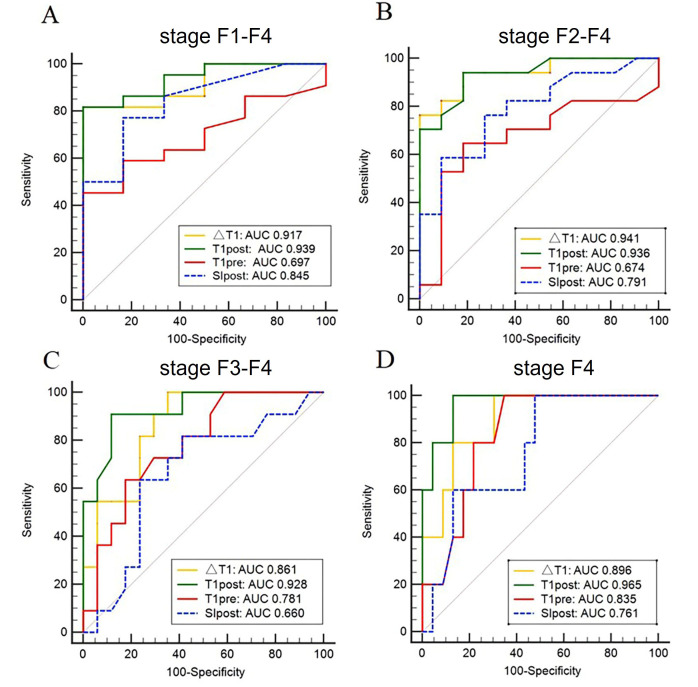
Receiver Operating Characteristic (ROC) curves of T1_pre_, T1_post_, ΔT1%, and SI_post_ for diagnosing (**A**) stages F1–F4, (**B**) stages F2–F4, (**C**) stages F3-F4, and (**D**) stage F4. T1_pre_, precontrast T1 relaxation time; T1_post_, postcontrast T1 relaxation time; ΔT1%, reduction rate of T1 relaxation time; SI_post_, postcontrast signal intensity.

The cut-off values, sensitivity, specificity, and corresponding AUCs of T1_pre_, T1_post_, ΔT1%, and SI_post_ for differentiating fibrosis stages are summarized in Table **2** and Fig. **4**. The AUCs of T1_pre_, T1_post_, ΔT1% and SI_post_ were 0.697, 0.939, 0.917, and 0.845, respectively, for diagnosing stages F1-F4; 0.674, 0.936, 0.941, and 0.791, respectively, for diagnosing stages F2-F4; 0.781, 0.928, 0.861, and 0.660, respectively, for diagnosing stages F3-F4; and 0.835, 0.965, 0.896, and 0.761, respectively, for diagnosing stage F4.

The AUCs of T1_post_ and ΔT1% were better than those of SI_post_ for diagnosing stages F2–F4 (0.936, 0.941 *vs.* 0.791, *p* = 0.043, 0.038, respectively), stages F3-F4 (0.928, 0.861 *vs.* 0.660, *p* = 0.003, 0.028, respectively) and stages F4 (0.965, 0.896 *vs.* 0.761, *p* = 0.021, 0.049, respectively). The AUCs of T1_post_ and ΔT1% were higher than those of T1_pre_ for diagnosing stages F1–F4 (0.939, 0.917 *vs.* 0.697, *p* = 0.013, 0.045, respectively) and stages F2–F4 (0.936, 0.941 *vs.* 0.674, *p* = 0.018, 0.003, respectively). The performances of T1_post_ and ΔT1% were not significantly different across all fibrosis stages (all *p* > 0.05).

### 3.4 Correlations Between T1_pre_, T1_post_, and ΔT1% and Hepatocyte Transporter Expression

**Fig. (5):**
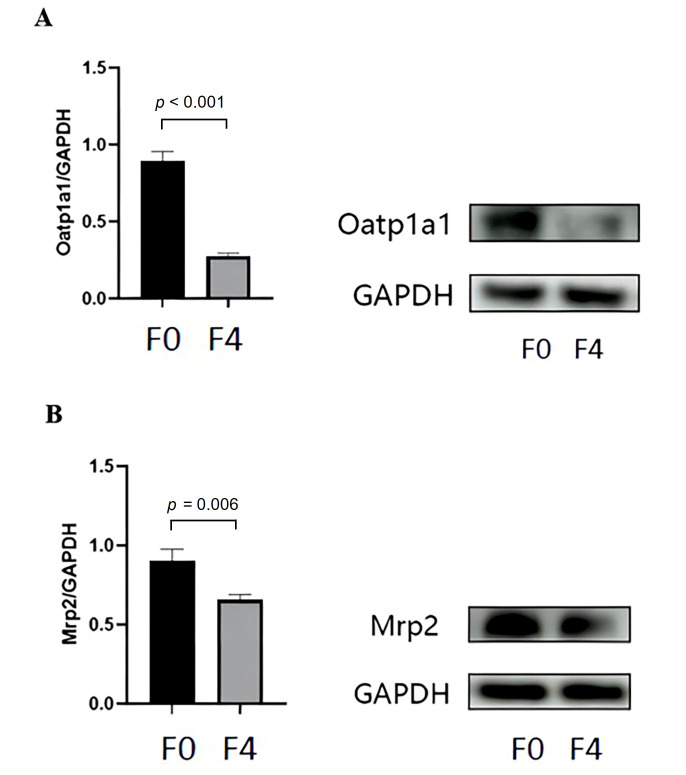
The expression levels of (**A**) Oatp1a1 and (**B**) Mrp2 at stages F0 and F4 were measured *via* Western blotting. Oatp1a1, organic anion transporting polypeptide 1a1; Mrp2, multidrug resistance-associated protein 2.

We detected the protein expression levels of Oatp1a1 and Mrp2 in stages F0 and F4 *via* western blotting (Fig. **5**). The Western blot results revealed that the expression levels of Oatp1a1 and Mrp2 were lower in stage F4 than in stage F0 (all *p* < 0.05). The expression levels of Oatp1a1 and Mrp2 in stages F0 and F4 were significantly negatively correlated with T1_post_ (r = −0.859, 95% CI: −0.990 to −0.627, *p =* 0.001; r = −0.697, 95% CI: −0.933 to −0.233, *p* = 0.017) and ΔT1% (r = 0.891, 95% CI: 0.635−0.988, *p* < 0.001; r = 0.685, 95% CI: 0.406 to 0.961, *p* = 0.020). However, no correlation was found between T1_pre_ and the expression levels of Oatp1a1 and Mrp2 (r = −0.517, 95% CI: −0.821 to 0.284, −0.578, 95% CI: −0.976 to −0.011, *p* = 0.104, 0.062, respectively) (Table **3**).

## 4 DISCUSSION

Distinguishing the various stages of liver fibrosis is vital in clinical applications, as early-stage liver fibrosis is sometimes reversible with timely intervention or treatment [22]. In our study, Gd-BOPTA-enhanced T1 mapping *via* the B1 inhomogeneity-corrected VFA method was effective in assessing the stage of liver fibrosis, providing a noninvasive and reliable method for its diagnosis.

In this study, we first compared the differences in T1_pre_, T1_post_, and ΔT1% across various stages of liver fibrosis. We found that as liver fibrosis progressed, T1_post_ tended to increase, whereas ΔT1% tended to decrease. These findings are consistent with the results of previous studies [23]. In diffuse liver disease, hepatocyte injury results in increased edema and ECM remodeling, which lengthens the T1 relaxation time and tends to shorten the rate of change in the T1 relaxation time [24, 25]. Moreover, we found no significant differences in T1_pre_ between different fibrosis stages. Li *et al.* [26] considered native T1 mapping to be a reliable method for noninvasively assessing liver fibrosis. However, some previous studies have reported no significant correlation between precontrast T1 relaxation times and liver fibrosis, and precontrast T1 relaxation times may be influenced by several factors in addition to the fibrosis stage [27]. On the basis of these controversial studies, we consider that T1_pre_ may not be an accurate indicator for assessing liver fibrosis.

The results of our study indicated that both T1_post_ and ΔT1% have good diagnostic performance for staging liver fibrosis. The AUCs of T1_post_ and ΔT1% were greater than those of SI_post_ for diagnosing stages F2–F4, F3-F4, and F4. It has been reported that the hepatocyte phase signal intensity of the liver parenchyma on the basis of Gd-BOPTA-enhanced MRI can be used to evaluate liver fibrosis [28]. However, the signal intensity often varies at different time points due to MRI technical factors [29]. Our results confirmed the advantage of Gd-BOPTA-enhanced T1 mapping over conventional signal intensity measurements for assessing liver fibrosis, which aligns with the conclusions of Pan *et al.* [16] and Sheng *et al.* [23]. As an absolute value, the T1 relaxation time was directly related to the concentration of the contrast agent, which was superior to the signal intensity from the gray images [29]. Currently, gadoxetic acid-enhanced (Gd-EOB-DTPA, Primovist, Bayer Healthcare) and Gd-BOPTA are widely used as hepatocyte-specific contrast agents in the diagnosis of liver diseases, as they are both taken up by functional hepatocytes and excreted into the bile and kidneys [28, 30]. Notably, the hepatobiliary phase based on Gd-EOB-DTPA may overlap with its late dynamic phase, which may lead to difficulties in image reading and fibrosis analysis, whereas the hepatobiliary phase of Gd-BOPTA overcomes this problem [31]. In the future, we plan to compare the diagnostic values of Gd-BOPTA-enhanced T1 mapping and other imaging techniques (*e.g*., apparent diffusion coefficient values) for staging liver fibrosis.

The protein expression levels of Oatp1a1 and Mrp2 were compared in stage F0 and stage F4 rats *via* western blotting, and the results showed that their expression levels tended to decrease as cirrhosis progressed. Gd-BOPTA in the rat liver is taken up by hepatocytes *via* Oatp1a1, Oatp1a4, and Oatp1b2 and is subsequently excreted into the bile duct *via* Mrp2 [32]. As liver fibrosis progresses, particularly in the cirrhosis stage, the number of functional hepatocytes decreases, which leads to lower expression levels of Oatps and Mrp2 [33]. Additionally, we found that the expression levels of hepatocyte transporters in stages F0 and F4 were negatively and positively correlated with T1_post_ and ΔT1%, respectively, indicating that Gd-BOPTA-enhanced T1 mapping can be an effective biomarker associated with hepatocyte transporter expression in cirrhosis. Hepatic fibrosis is a diffuse liver disease characterized by a decrease in the number of normal liver cells and a weakening of the ability to absorb specific contrast agents, leading to changes in the T1 relaxation time in the hepatobiliary phase [34]. Moreover, liver fibrosis is often accompanied by changes in other tissues, such as inflammatory edema, steatosis, and iron deposition in liver cells. These changes can also affect the T1 relaxation time of the liver [23]. Results from Sheng *et al.* [23] showed that, compared with necrotizing inflammatory activity and iron overload, fibrosis was the only factor independently predicted by postcontrast and ΔT1% relaxation times, which suggests that hepatocyte-specific enhanced T1 mapping may be a reliable method for staging liver fibrosis.

Among the various T1 mapping methods, the most commonly used one is the modified look-locker inversion recovery sequence, which, to some extent, optimizes and shortens the scanning time [35] but still cannot meet the needs of whole-liver scanning and can only scan a high-resolution liver slice in one breath hold [36]. Our study used B1 inhomogeneity-corrected VFA to obtain T1 mapping images. This technology has become available by applying the B1 mapping pulse sequence before T1 mapping, which involves using two or more different angles of imaging to calculate the T1 values of tissues and organs [37]. This sequence has high spatial resolution and fast imaging speed. Moreover, it also addresses the issue of limited liver coverage due to the long acquisition time [17, 38], thereby improving the accuracy of our T1 mapping parameter results. Since image quality is crucial for accurate T1 mapping, the advanced denoising techniques would complement our T1 mapping methodology [39]. The ensemble approaches for medical diagnostics may offer new perspectives for combining T1_pre_, T1_post_, and ΔT1% measurements and achieving superior performance in fibrosis assessment [40].

## 5 STUDY LIMITATIONS

There were a few limitations of the present study. First, CCl_4_-induced liver fibrosis in animal models, and patients with liver fibrosis had differences in their pathological mechanisms. The results of this study need to be further confirmed in patients, especially those with liver fibrosis caused by hepatitis B or C. Second, the sample size was relatively small. Third, we only compared the expression levels of hepatocyte transporters between stages F0 and F4 *via* the Western blot analysis; future work will include these intermediate stages to better understand the relationships between transporter changes and imaging throughout fibrosis progression.

## CONCLUSION

In this study, T1_post_ and ΔT1% were significantly correlated with liver fibrosis stages and demonstrated superior diagnostic performance in differentiating stages F2–F4, F3–F4, and F4. These imaging biomarkers also showed significant correlations with hepatocyte transporter expression. Gd-BOPTA-enhanced T1 mapping via the B1 inhomogeneity-corrected VFA method shows promise as a potentially accurate and effective method for the quantitative assessment of liver fibrosis.

## Figures and Tables

**Table 1:** T1_pre_, T1_post_, and ΔT1% at different liver fibrosis stages.

**Stage**	**Number**	**T1_pre_ (ms)**	**T1_post_ (ms)**	**ΔT1%**
**F0**	6	1006.33±13.88	484.83±14.40	51.82±1.37
**F1**	5	1018.4±21.65	510.8±24.77	49.85±2.05
**F2**	6	998.34±43.09	532±29.70	46.65±3.35
**F3**	6	1025.33±19.53	555±32.48	45.88±2.85
**F4**	5	1037.6±14.43	594.4±27.26	42.72±2.39

**Notes:** T1_pre_, precontrast T1 relaxation time; T1_post_, postcontrast T1 relaxation time; ΔT1%, reduction rate of the T1 relaxation time.

**Table 2:** Diagnostic performance of T1_pre_, T1_post_, ΔT1%, and SI_post_ for diagnosing fibrosis stages.

**Parameters**	**stage F1–F4**	**stage F2–F4**	**stage F3-F4**	**stage F4**
AUC	-	-	-	-
ΔT1%	0.917 (0.749-0.987)	0.941 (0.783-0.995)	0.861 (0.678-0.962)	0.896 (0.721-0.979)
T1_post_	0.939 (0.780-0.994)	0.936 (0.775-0.993)	0.928 (0.764-0.991)	0.965(0.818-0.999)
T1_pre_	0.697 (0.495-0.855)	0.674 (0.472-0.838)	0.781 (0.585-0.914)	0.835(0.647-0.947)
SI_post_	0.845(0.658-0.953)	0.791 (0.597-0.921)	0.660 (0.458-0.827)	0.761(0.563-0.901)
Sensitivity (%)	-	-	-	-
ΔT1%	81.82	76.47	100.00	100.00
T1_post_	81.82	94.12	90.91	100.00
T1_pre_	45.45	64.71	63.63	100.00
SI_post_	77.27	58.82	81.82	95.65
Specificity (%)	-	-	-	-
ΔT1%	100.00	100.00	64.71	69.57
T1_post_	100.00	81.82	88.24	86.96
T1_pre_	100.00	81.82	82.35	65.22
SI_post_	83.33	90.91	58.82	52.17
Cut-off value	-	-	-	-
ΔT1%	50.05	46.73	49.85	46.40
T1_post_	505	505	543	550
T1_pre_	1021	1019	1021	1019
SI_post_	394	382	391	391

**Notes:** T1_pre_, precontrast T1 relaxation time; T1_post_, postcontrast T1 relaxation time; ΔT1%, reduction rate of T1 relaxation time; SI_post_, postcontrast signal intensity; AUC, area under the curve.

**Table 3:** Correlation between T1_pre_, T1_post_, and ΔT1% and hepatocyte transporter expression.

	**T1_pre_**	**T1_post_**	**ΔT1%**
**Oatp1a1/GAPDH**	-	-	-
**r value**	-0.517 (-0.821 to 0.284)	-0.859 (-0.990 to -0.627)	0.891 (0.635-0.988)
***p* value**	0.104	0.001	<0.001
**Mrp2/GAPDH**	-	-	-
**r value**	-0.578(-0.976 to -0.011)	-0.697 (-0.933 to -0.233)	0.685 (0.406 to 0.961)
***p* value**	0.062	0.017	0.020

Notes: T1_pre_, precontrast T1 relaxation time; T1_post_, postcontrast T1 relaxation time; ΔT1%, reduction rate of the T1 relaxation time.

## Data Availability

The data and supportive information are available within the article.
